# Linear atrophoderma of Moulin is due to the decreased dermal thickness

**DOI:** 10.1111/srt.13175

**Published:** 2022-06-10

**Authors:** Li‐wen Zhang, Cong‐hui Li, Li‐xin Fu, Pei‐mei Zhou, Hui‐min Meng, Xue Shen, Jing Nie, Tao Chen

**Affiliations:** ^1^ Department of Dermatovenereology Chengdu Second People's Hospital Chengdu Sichuan China

Dear Editor,

Linear atrophoderma of Moulin (LAM) is a rare cutaneous disease characterized by the unilateral hyperpigmented atrophoderma following Blaschko's lines without evident inflammation and sclerosis. Usually, it begins in childhood or adolescence without long‐term progression. The etiology of LAM and the atrophic reasons are still unknown. We reported 4 cases of LAM and evaluated the atrophy by high‐frequency ultrasound (MD‐300S II, MaiDa, China) to further reveal the development of LAM. We suggested that the clinical atrophy in most LAM is due to the decreased dermal thickness and a few severe cases might also involve subcutis.

**FIGURE 1:**
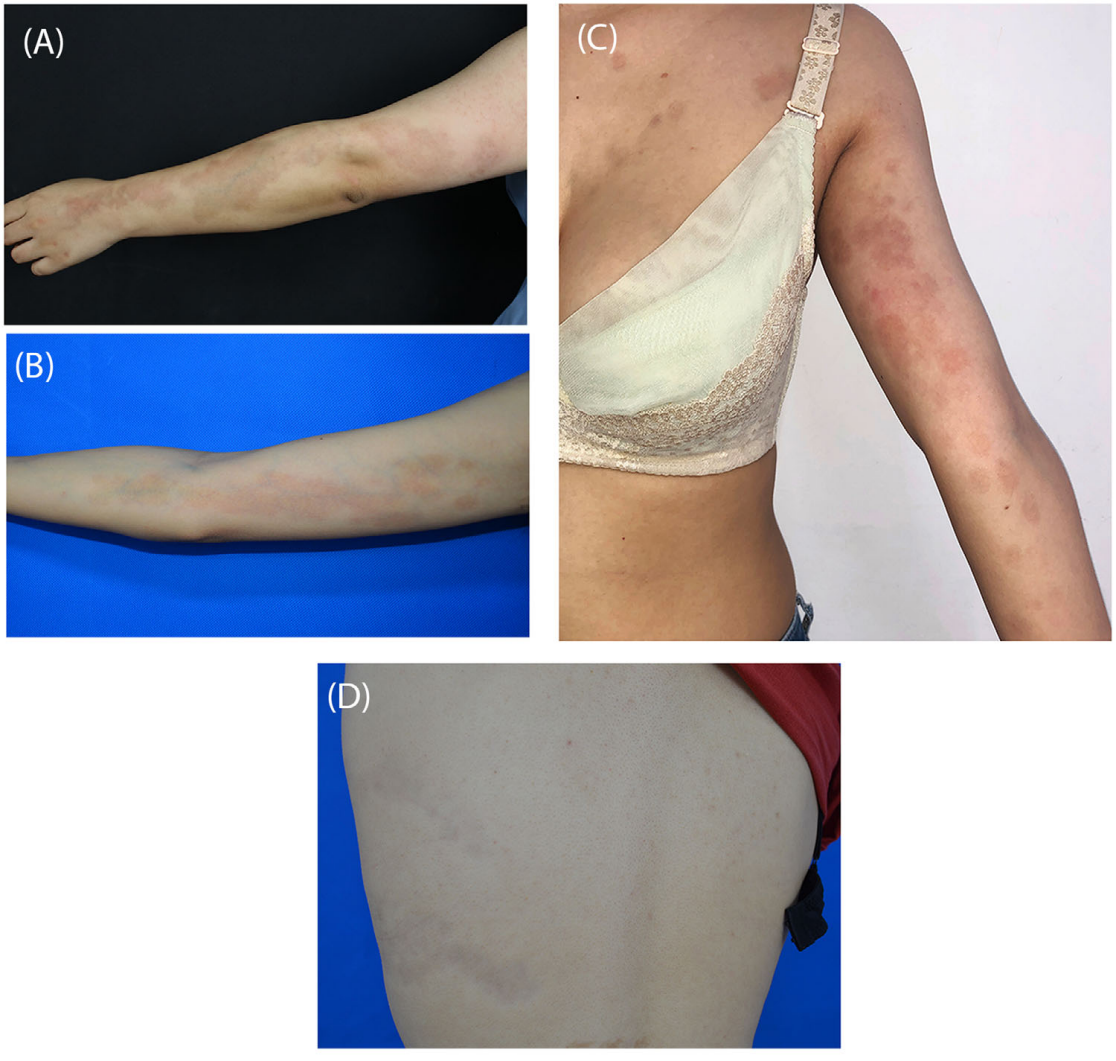
Clinical images: unilateral linear hyperpigmented atrophic patches along Blaschko's lines (a: Case 1; b: Case 2; c: Case 3; d: Case 4)

**FIGURE 2:**
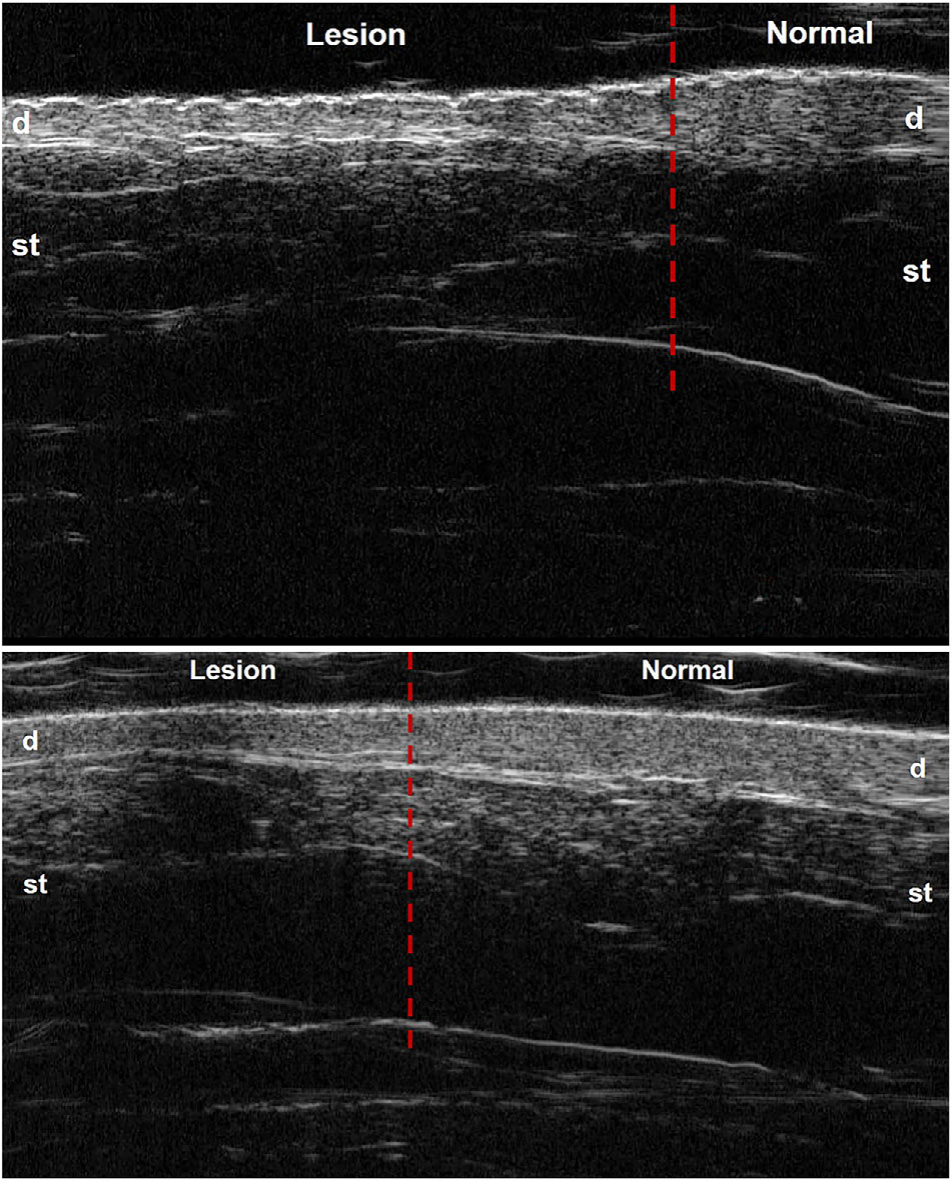
Ultrasonic images (Frequency: 50 MHz): a. The dermal and subcutaneous thickness decreased in Case 1; b. The decreased dermal thickness and normal subcutaneous tissue in Case 2. (d, derma; st, subcutaneous tissue)

**Case 1**. A 24‐year‐old female presented with a 15‐year history of asymptomatic, unilateral, atrophic, brown patches on her left arm along Blaschko's lines (Figure 1a). The lesions aggravated gradually in the first few years, then became static. Ultrasonic examination showed decreased dermal thickness with hyperechogenicity and decreased subcutaneous thickness with normal echogenicity compared with the normal skin (Figure 2a).

**Case 2**. A 23‐year‐old female presented with a 10‐year history of asymptomatic, unilateral, atrophic, brown patches on her left arm and prothoracic regions followed Blaschko's lines (Figure 1b). Ultrasonic examination showed decreased dermal thickness with normal echogenicity and normal subcutaneous thickness and echogenicity in the lesions (Figure 2b).

**Case 3**. A 15‐year‐old girl presented with a 10‐year history of asymptomatic, unilateral, atrophic, brown patches on her right arm followed Blaschko's lines (Figure 1c). Ultrasonic examination results and histopathologic manifestations were similar to Case 2.

**Case 4**. A 16‐year‐old girl presented with an 11‐year history of asymptomatic, unilateral, atrophic, brown patches on her right arm followed Blaschko's lines (Figure 1d). Ultrasonic examination results and histopathologic manifestations were similar to Case 2.

The clinical and ultrasonic features of 4 patients were summarized in Table 1. No signs of sclerosis or induration were noted in four cases. They denied significant medical or family history. They got complete blood cell count and the spectrum of antinuclear antibodies, which results were unremarkable. The histopathology revealed hyperpigmentation in the epidermal basal layer and mild perivascular lymphocytic infiltration in the dermis. The four patients were diagnosed with LAM based on the clinical and histopathological findings.

**TABLE 1:** The clinical and ultrasonic features of 4 patients with linear atrophoderma of Moulin

Case	Sex	Age, years	Course, years	Locations	Ultrasonic features	Prognosis
1	F	24	15	Left arm	Decreased dermis with hyperechogenicity Decreased subcutaneous tissue with normal echogenicity	Static lesions without relief
2	F	23	10	Left arm and prothoracic regions	Decreased dermis with normal echogenicity Normal subcutaneous thickness and echogenicity	Static lesions without relief
3	F	15	10	Right arm	Decreased dermis with normal echogenicity Normal subcutaneous thickness echogenicity	Static lesions without relief
4	F	23	13	Left neck, chest and back	Decreased dermis with normal echogenicity Normal subcutaneous thickness echogenicity	Static lesions without relief

Moulin first described LAM in 1992,^1^ and there were more than 40 relevant literature reports so far. The etiology and pathogenesis of LAM are still unclear. All patients that have been reported were sporadic, and it has been postulated that LAM may be mosaic from a postzygotic autosomal mutation in genes due to the linear distribution following Blaschko's lines.^2^ Another hypothesis pointed out that LAM was an autoimmune disease. These three diseases were a spectrum of disorders rather than unique entities because LAM had many clinical and histologic similarities with atrophoderma of Pasini and Pierini and linear morphea and antinuclear antibody abnormalities were revealed in several cases LAM.^3^, ^4^

There was no significant difference in the ratio of males to females, although the four patients we reported were all female. LAM usually presented unilateral, and bilateral presentation was rare. Lesions most commonly involved the trunk and upper limb, followed by the lower limb, and rarely in the faciocervical regions.^2^ The atrophic lesions aggravated gradually in the first few years without obvious inflammatory reaction, then became static and non‐sclerosis. Histopathologically, LAM was nonspecific and manifested hyperpigmentation in the epidermal basal layer and a mild perivascular lymphocytic infiltration with or without slightly altered collagen fiber.^2^

The causes of clinical atrophy in LAM were still controversial. Norisugi reported a case of LAM at the early stage and suggested the possible reason might be a reduction of the subcutaneous tissue.^5^ Li described another patient with a long course that showed decreased dermal thickness and normal subcutis.^6^ In our patients, four cases presented decreased dermal thickness and one of them also showed decreased subcutaneous thickness. These variations may be due to different courses and severity of disease, like morphea. Under ultrasound, increased dermal thickness with decreased echogenicity and increased subcutaneous tissue echogenicity were be manifested in the early active morphea.^7^ However, the ultrasonic manifestations showed decreased dermal and subcutaneous thickness in the late atrophic lesion of morphea.^7^ In our case, the clinical atrophy in most LAM is due to the decreased dermal thickness. These different ultrasonic findings can be used to distinguish LAM between morphea.

In conclusion, high‐frequency ultrasound is a useful noninvasive evaluation tool for atrophic cutaneous disease. The clinical atrophy in LAM is primarily due to the decreased dermal thickness, but it is usually not evident at the early stage, and a few severe cases might also involve subcutis. This hypothesis needs further larger clinical studies to prove.
